# Comparative efficacy of different estrus synchronization protocols on estrus induction response, fertility and plasma progesterone and biochemical profile in crossbred anestrus cows

**DOI:** 10.14202/vetworld.2015.1310-1316

**Published:** 2015-11-21

**Authors:** A. J. Dhami, B. B. Nakrani, K. K. Hadiya, J. A. Patel, R. G. Shah

**Affiliations:** Department of Animal Reproduction, Gynaecology and Obstetrics, College of Veterinary Science and Animal Husbandry, Anand Agricultural University, Anand-388 001, Gujarat, India

**Keywords:** anestrus, conception rate, cow, estrus induction, fertile estrus induction interval,treatment protocols

## Abstract

**Aim:**

To evaluate estrus induction response and fertility including plasma progesterone and biochemical profile following use of three standard hormonal protocols in anestrus crossbred cows.

**Materials and Methods:**

The study was carried out on 40 true anestrus and 10 normal cyclic cows. 10 anestrus cows each were treated with standard intravaginal controlled internal drug release (CIDR) device, Ovsynch (GPG) protocol, and Norgestomet ear implant with fixed-time artificial insemination (FTAI). 10 anestrus cows were kept as untreated control while 10 cows exhibiting the first estrus within 90 days postpartum without any treatment served as normal cyclic control. Blood samples were obtained from treated cows on day 0, 7, 9 (AI) of treatment and day 21 post-AI, and from control groups on the day of AI and day 21 post-AI for estimation of plasma progesterone, protein, cholesterol, calcium, and inorganic phosphorus profile.

**Results:**

The use of CIDR, Ovsynch, and Norgestomet ear implant protocols resulted in 100% estrus induction with conception rates at induced estrus of 60%, 50%, and 50%, and the overall of three cycles as 80%, 80%, and 70%. In untreated anestrus control (n=10), only three cows exhibited spontaneous estrus within 90 days of follow-up and conceived giving the first service and overall conception rates of 66.66% and 30.00%, respectively. In normal cyclic control (n=10), the conception rates at first and overall of three cycles were 50% and 80%. The overall mean plasma progesterone (P_4_) concentrations in anestrus cows studied on day 0 (initiation), 7 (prostaglandin injection and/or removal of implant), 9 (FTAI) of treatment and on day 21 post-AI revealed that the values on day 7 and 21 were significantly (p<0.01) higher than other two periods in all three groups. The concentrations were significantly (p<0.05) higher in conceived than non-conceived group on day 21 post-AI in CIDR (4.36±0.12 vs. 1.65±0.82 ng/ml) and Ovsynch (4.85±0.62 vs. 1.59±0.34 ng/ml), but not in Norgestomet ear implant (4.50±0.53 vs. 3.02±1.15 ng/ml) or normal cyclic group (5.39±0.67 vs. 3.13±0.37 ng/ml). The cholesterol and protein levels were significantly higher, but not the calcium and phosphorus, in normal cyclic control than in anestrus groups. The influence of treatment days and pregnancy status was not significant for any of the biochemical constituents in any of the groups.

**Conclusion:**

Ovsynch and/or CIDR synchronization protocol can be effectively used to improve fertility up to 80% in anestrus cows, as compared to 30% in anestrus control, combined with plasma progesterone to delineate the reproductive status before and after treatment.

## Introduction

Genetic upgradation of indigenous cattle population through crossbreeding is the only immediate approach to meet the challenges of milk production demand in India. The optimum reproductive efficiency of these animals is equally important for economic productivity. Infertility is one of the pathological conditions qualified as disease of production. It is widespread in modern farming, especially in crossbred animals. Infertility negatively affects productivity and return on investment of the farmers. Anestrus forms the major condition constituting about 2/3^rd^ of the infertility problems in crossbred cattle [1]. Various hormonal preparations and protocols are being practiced by the field veterinarians to treat postpartum anestrus, the most prevalent reproductive problem, in dairy animals, but the results are inconsistent [2-4]. Hormonal therapies have good therapeutic value to enhance reproductive efficacy in infertile animals only with good nutritional status [2,3,5]. The variable results obtained following hormonal treatments by different workers may be largely due to nutritional status, faulty management, ovarian changes, endocrine events, and even uterine infection. Use of hormonal protocols like Ovsynch, controlled internal drug release (CIDR) device and Norgestomet ear implant can be helpful in inducing and synchronizing estrus and getting better conception rate in anestrus dairy bovines with lesser number of services per conception and making acyclic ones to cycle normally, thereby achieving ideal inter-calving interval of 12-13 months [1,2,6].

The progesterone hormone is responsible for stimulation of cyclicity, follicular development and also for maintenance of pregnancy. The plasma protein, cholesterol and minerals profile denote the nutritional status of animals and are related with their fertility [5,7]. The cholesterol being precursor of steroid hormones play an important role in steroidogenesis; while calcium tones up the genitalia, and protein and inorganic phosphorus are involved at the cellular level in metabolic processes. These hormonal and nutritional profiles are being disturbed by many metabolic and environmental factors and hamper normal physiology of the animal body [7].

The comparative studies involving the use of different estrus induction/synchronization protocols at a time under an identical environment in crossbred cows are, however, meager and are mostly based on clinical response only without plasma progesterone or biochemical evaluation [7-9]. Hence, this study was planned to evaluate the comparative efficacy of CIDR, Ovsynch and Norgestomet ear implant protocols in anestrus crossbred cows under field conditions in terms of estrus induction response, fertility enhancement, and their influence on plasma progesterone and biochemical profile.

## Materials and Methods

### Ethical approval

The prior approval from the Institutional Animal Ethics Committee was obtained for use of farmers animals in this study.

### Selection and treatment of animals

This study was carried out during November, 2013 to March, 2014 under middle Gujarat agro-climatic condition. 40 postpartum (>90 days) anestrus crossbred cows and 10 normal cyclic cows of the average body condition score were selected from villages of Amul and Panchamrut milk-shed areas of Gujarat. The cows were screened gynaeco-clinically for their reproductive status. Detailed history and rectal palpation findings were recorded. Anestrus cows were confirmed by palpating small smooth inactive ovaries per rectum twice 10 days apart. All the selected cows were dewormed using ivermectin, 100 mg S/C and were supplied with multi-mineral boluses one bolus daily for 7 days. They were randomly subjected to following three estrus induction/synchronization protocols (*viz*., CIDR, Ovsynch and Norgestomet ear implant, n=10 each) with fix-timed artificial insemination (FTAI) [2,5,7,10].

### Treatment protocols

**Figure-1:**
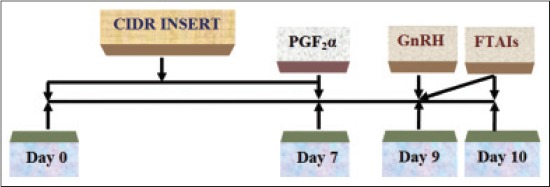
CIDR protocol of estrus synchronization.

In 10 true anestrus crossbred cows, CIDR (1.38 g of progesterone in the silastic coil, Pfizer Animal Health, Mumbai) was inserted intravaginally on day 0. It was removed on day 7 together with I/M injection of prostaglandin F2α (PGF_2_α) 25 mg (dinoprost tromethamine,). Injection GnRH 10 µg (buserelin acetate,) was administered I/M on day 9 and FTAIs were performed twice on day 9 and 10, as shown in Figure-1.

**Figure-2:**
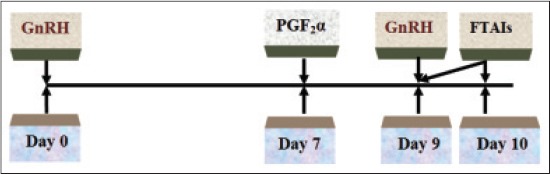
Ovsynch protocol of estrus synchronization.

10 true anestrus cows under Ovsynch protocol were administered intramuscularly with injection buserelin acetate - GnRH 20 µg on day 0, injection PGF_2_α 25 mg on day 7, and second injection of GnRH 10 µg on day 9 followed by FTAIs twice on day 9 and 10 (Figure-2).

**Figure-3:**
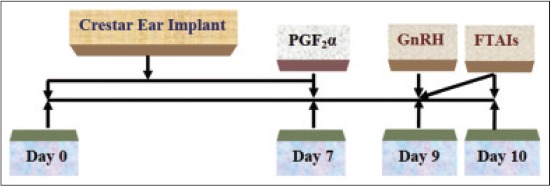
Norgestomet ear implant protocol of estrus synchronization.

In another group of 10 anestrus cows, crestar implant (containing 3.3 mg norgestomet, Intervet India Pvt. Ltd.) was inserted S/C in the outer face of the ear-base together with 2 ml Crestar injection I/M (injection containing 3 mg norgestomet and 5 mg estradiol valerate) on day 0. The implant was removed on day 7 together with I/M injection of 25 mg PGF_2_α dinoprost tromethamine and injection buserelin acetate 10 µg I/M was given on day 9 followed by FTAIs twice at 0 and 24 h later (Figure-3). Signs of estrus and rectal palpation findings were recorded for animals of all the groups at AI.

Another 10 anestrus cows were kept as anestrus control without hormone therapy and 10 normal cyclic cows that expressed spontaneous estrus within 90 days postpartum and inseminated served as normal cyclic control group. Cows in spontaneous or induced estrus were inseminated using good quality frozen-thawed semen. Animals detected in estrus subsequent to FTAI were re-inseminated up to two cycles and in non-return cases pregnancy was confirmed rectum 60 days of last AI.

### Blood sampling

All the hormonally treated/untreated true anestrus and normal cyclic cows were studied for their reproductive status and plasma progesterone and biochemical profile. For this, jugular blood samples were collected in heparinized vacutainers 4 times from true anestrus animals, i.e., on day 0 - just before treatment (on diagnosis), on day 7 - at the time of PGF_2_α administration, on day 9 - induced estrus/FTAI (FTAI done twice 24 h apart, i.e., on day 9 and 10 after initiation of treatment) and on day 21 post-AI. Blood sampling for control groups was done on the day of spontaneous estrus if any, and on day 21 post-AI. The samples were centrifuged at 3000 rpm for 15 min and plasma separated out was stored deep frozen at −20°C with a drop of merthiolate (0.1%) until analyzed.

### Plasma assay

Plasma progesterone profile was estimated using the standard radio-immuno-assay (RIA) technique of Kubasic *et al*. [11]. Labeled antigen (I^125^), antibody coated tubes and standards were procured from Immunotech, France. The sensitivity of the assay was 0.1 ng/ml. The intra-and interassay coefficients of variation were 5.4% and 9.1%, respectively. The concentrations of plasma total protein, total cholesterol, calcium and inorganic phosphorus were determined by standard procedures and assay kits procured from Analytical Technologies Pvt. Limited, Baroda, on chemistry analyzer.

### Statistical analysis

The data on estrus response, conception rate (by Chi-square test) and plasma profiles of progesterone and biochemical constituents were analyzed statistically through analysis of variance [12] using online SAS software version 20.00.

## Results and Discussion

### Estrus induction and conception rates

All the cows (100%) under CIDR, Ovsynch, and Norgestomet ear implant protocols exhibited induced estrus with varying intensity similar to normal cyclic control group within 42-72 h from the time of PGF_2_α injection. The conception rates obtained at induced estrus in cows under these three protocols were 60.00%, 50.00%, and 50.00%, respectively, with corresponding overall pregnancy rates of three cycles as 80.00%, 80.00%, and 70.00%. Results were better with CIDR and Ovsynch protocols as compared to Norgestomet ear implant (Table-1). In untreated anestrus control group, only 3 out of 10 cows exhibited spontaneous estrus within 90 days of follow-up and two conceived at first AI (CR, 66.66%) and third at 3^rd^ AI giving overall pregnancy rate of only 30.00% (3/10). In the normal cyclic control group (n=10), the conception rates at the first cycle and overall of three cycles were 50.00% and 80.00%, respectively, with mean service period of 98.77±6.84 days (Table-1).

**Table-1:** Effects of various estrus induction/synchronization protocols on estrus induction response, estrus induction intervals, and conception rates in anestrus crossbred cows.

Treatment groups	No.	Estrus induction response (%)	PG injection to estrus interval (h)	Conception rate (%)				PG injection to fertile estrus interval (days)

				Induced/first estrus	Second cycle	Third cycle	Overall of three cycles	
CIDR	10	100.00 (n=10)	58.27±1.93	60.00 (6/10)	25.00 (1/4)	33.33 (1/3)	80.00 (8/10)	12.44±3.86 (n=8)
Ovsynch	10	100.00 (n=10)	64.13±1.33	50.00 (5/10)	40.00 (2/5)	33.33 (1/3)	80.00 (8/10)	12.89±4.31 (n=8)
Norgestomet ear implant	10	100.00 (n=10)	54.00±1.17	50.00 (5/10)	20.00 (1/5)	25.00 (1/4)	70.00 (7/10)	11.78±3.81 (n=7)
Untreated anestrus control	10	30.00 (n=3)	-	66.66 (2/3)	-	100.00 (1/1)	30.00 (3/10)	149.52±5.67* (n=3)
Normal cyclic control	10	100.00 (n=10)	-	50.00 (5/10)	40.00 (2/5)	33.33 (1/3)	80.00 (8/10)	98.77±6.84* (n=8)

Figures in parentheses indicate number of animals/observations,

* Service period/days open. PG=Prostaglandin, CIDR=Controlled internal drug release

The mean estrus induction intervals of 58.27±1.93, 64.13±1.33, and 54.00±1.17 h observed in cows under CIDR, Ovsynch and Norgestomet ear implant protocols compared favorably with the previous reports in anestrus cows [7,13,14] and buffaloes [3,5] using such types of protocols. Comparatively shorter interval was however reported by others [6] in heifers and multiparous cows. Chaudhari *et al*. [2] reported this interval to be much shorter as 25.41±0.94, 21.95±0.20 and 22.68±1.46 h using Crestar, Crestar + 500 IU PMSG, and Crestar + Receptal in Kankrej heifers. Following removal of implant resumption of follicular development and maturation is due to the flux of the gonadotropin from the pituitary gland. Although behavioral estrus in case of Norgestomet ear implant was observed because of direct effect of both exogenously administered estradiol and the high endogenous estradiol on the hypothalamus [15].

The first service conception rate of 50.00% with Norgestomet ear implant in the present study is comparable with the earlier result of Nak *et al*. [6] as 41.40% in anestrus heifers. The findings with CIDR and Ovsynch protocols are also in line with Patel *et al*. [7] as 50.00% and 30.00% in anestrus crossbred cows, respectively, and Bhoraniya *et al*. [10] as 66.66% and 33.33% overall conception rate in anestrus Kankrej cows with same protocols. Ozyurtlu *et al*. [16] reported overall conception rates of 44.00% and 53.85% in Norgestomet and PRID groups, respectively, which are relatively lower than the present findings with Norgestomet ear implant and CIDR. Relatively inferior pregnancy rate as 33.33% was also reported by Chaudhari *et al*. [2] with Norgestomet ear implant. Lower first service conception rates of 40.00% and 30.00% [7], and 36.84% and 29.41% [9], respectively, with CIDR and Ovsynch protocols, are also documented by others.

With Ovsynch and Norgestomet ear implant, Nak *et al*. [6] reported overall conception rate of 42.18% and 29.60% in non-cycling cows and 44.07% and 41.4% for heifers. Martinez *et al*. [17] also reported that the addition of progestin to the Cosynch or Ovsynch regimen resulted in significantly improved pregnancy rates in heifers but not in cows. El-Zarkouny *et al*. [4] reported that anestrus dairy cow treated with Ovsynch plus CIDR had a higher pregnancy rate (64%) than anestrus cows treated with Ovsynch alone (27%). However, cycling cows receiving Ovsynch plus CIDR had a pregnancy rate similar to that of cycling cows receiving Ovsynch alone. Stevenson *et al*. [18] reported that pregnancy outcomes showed larger increases when cows were treated with Ovsynch plus CIDR than with Ovsynch alone because more anestrus cows conceived. However, our results showed similar results with CIDR and Ovsynch protocols but Norgestomet ear implant group showed less overall pregnancy outcomes than other two protocols. The reduced fertility at norgestomet induced estrus may be owing to the luteal dysfunction [19], which may be due to insufficient luteinizing hormone production following implant withdrawal [20]. Although better conception rate was obtained by Rentfrow *et al*. [13] in Synchro-Mate-B treated Brahman heifers (18.2%) and Singh *et al*. [21] in anestrus heifers and cows (40%).

Further, around 30% conception rates were obtained at the second and third cycle in anestrus cows induced to cycle, which is near to normal cycling cows (40%and 33%). This proved that all the protocols induced and synchronized the estrus and then established normal cyclicity in treated animals, resulting into conceptions in subsequent cycles like normal breeding cows. These observations further supported the previous observations on the use of similar protocols in anestrus cows and buffaloes by many workers [2,7,9,10,15,22].

Thus, estrus could be induced in true anestrus cows within 2-3 days from the day of PGF_2_α injection in each protocol and made them pregnant within a period of 10-12 days (95-100 days postpartum) in comparison to 149.52±5.67 days of service period recorded in untreated control group, indicating a huge curtailment (around 1.5-2.0 months) in the waiting period of anestrus animals to evince estrus and become pregnant. The pooled conception rates of three treatment protocols obtained (76.66%) in anestrus cows indicated the positive contributory role of handling the problem of acyclicity in cows, nearly at par with normal cyclic control cows (80.00%).

### Plasma progesterone profile

The mean levels of plasma progesterone recorded on day 0, 7, 9 (AI) of treatment and on day 21 post-AI in anestrus cows under CIDR, Ovsynch, and Norgestomet ear implant protocols, and on the day of AI and day 21 post-AI in the normal control group are presented in Table-2. The data show that the mean plasma progesterone (ng/ml) concentrations were low toward basal values on day 0, i.e., on the day of initiation of treatment in all three groups, suggesting that the animals were in anestrus phase. These levels, subsequently, rose significantly (p<0.01) to the peak values on day 7 (5.58±0.98, 4.10±0.78 and 1.92±0.23 ng/ml), particularly in animals under CIDR and Ovsynch protocols, i.e. just before implants were removed and PGF_2_α was injected. Thereafter, the mean progesterone levels dropped suddenly and significantly within 48 h of PGF_2_α injection and/or implant removal to the basal values coincident to induced estrus when FTAIs were done. These levels again increased significantly (p<0.01) on day 21 post-AI in all the groups (3.27±0.54, 3.22±0.64, and 3.76±0.65 ng/ml) due to estruses being ovulatory with development and maintenance of corpus luteum (CL) and the establishment of pregnancy in some animals. In normal cyclic control group also the mean plasma progesterone concentration was the lowest (0.43±0.17 ng/ml) on the day of spontaneous estrus/AI, which rose significantly (p<0.05) on day 21 post-AI (4.98±0.45 ng/ml) due to the establishment of pregnancy in four cows in that cycle.

**Table-2:** Plasma progesterone concentrations (ng/ml) in normal cyclic and anestrus cows on different days of treatment/AI under various estrus induction protocols.

Treatment groups	Pregnancy status	No.	Days from initiation of treatment/AI			

			Day 0	Day 7	Day 9 (AI)	Day 21 post-AI
CIDR	Conceived	6	2.19±0.51	5.88±1.27	0.64±0.15	4.36±0.12*
	Non-conceived	4	2.68±0.55	5.13±1.81	1.49±1.07	1.65±0.82
	Overall	10	2.38±0.36^ab^	5.58±0.99^c^	0.98±0.42^a^	3.27±0.54^b^
Ovsynch	Conceived	5	1.70±0.34	4.98±0.96	0.69±0.16	4.85±0.62*
	Non-conceived	5	2.38±0.55	3.22±1.18	1.60±0.34	1.59±0.34
	Overall	10	2.04±0.33^ab^	4.10±0.78^c^	1.14±0.23^a^	3.22±0.64^bc^
Norgestomet ear implant	Conceived	5	1.68±0.29	1.93±0.35	0.60±0.08	4.50±0.53
	Non-conceived	5	1.23±0.22	1.91±0.33	0.83±0.23	3.02±1.15
	Overall	10	1.45±0.19^ab^	1.92±0.23^b^	0.72±0.12^a^	3.76±0.65^c^
Normal cyclic control	Conceived	4	-	-	0.57±0.34	5.39±0.67
	Non-conceived	6	-	-	0.35±0.23	3.13±0.37
	Overall	10	-	-	0.43±0.17^a^	4.98±0.45^b^

* p<0.05; Means bearing uncommon superscripts within the row/column differ significantly (p<0.05). Day 0=Day of starting the treatment, Day 7=Administration of PG, Day 9=Fixed time AI, Day 21=Day 21 post-AI. Blood profile of untreated control cows is not shown in table due to only small number conceived (2 out of 10) in that group. PG=Prostaglandin, AI=Artificial insemination

The mean plasma progesterone concentrations in conceived and non-conceived groups in all three treatment protocols and in normal cyclic control group were found to be statistically similar on day 0, 7 and even on day 9 (AI), but on day 21 post-AI, the conceived cows had significantly higher mean plasma progesterone concentrations as compared to non-conceived ones only in CIDR (4.36±0.12 vs. 1.65±0.82 ng/ml) and Ovsynch (4.85±0.62 vs. 1.59±0.34 ng/ml) protocols (Table-2). These findings on plasma progesterone profile with respect to effect of CIDR and Ovsynch protocols and/or in normal cyclic group closely corroborated with the earlier observations in anestrus cows [7,9,10] and in anestrus buffaloes [3,5,23] under such protocols. The levels of plasma P_4_ on the day of beginning of treatment protocol helped delineate the reproductive and endocrine status of the animals and thereby predicting the possible response to the therapy. The higher plasma P_4_ recorded on day 21 post-AI in conceived cows of all the groups was due to establishment of pregnancy and maintenance of CL function, while significantly low yet variable plasma P_4_ noted on day 21 post-AI in non-conceived cows could be due to their return to next estrus at varying intervals on account of probable irregular or long cycle length, early embryonic mortality after day 17 or uncoordinated, unexplained hormonal changes in some of them. These findings corroborated with the observation of Nakrani *et al*. [5] using same three protocols in buffaloes and of Ayad *et al*.[24] using Norgestomet ear implant in cattle.

The mean plasma progesterone levels obtained on the day of initiation of CIDR and Ovsynch treatments in the present study corroborated with the earlier findings in zebu and crossbred cows [7,9,10,25]. Significant rise observed in plasma P_4_ profile on day 7 of treatments in the present study with CIDR and Ovsynch protocols (4.97±1.68 and 3.75±0.47 ng/ml) over initial (0 day) values, with sudden drop to almost basal values on induced estrus within 48-60 h after PG injection (Table-2), has also been reported earlier in anestrus cows [9,10,22] by employing CIDR and Ovsynch protocol. The apparently higher mean levels of progesterone found on day 21 post-AI in non-conceived cows covered under Norgestomet ear implant protocol and normal control group (3.02±1.15 and 3.13±0.37 ng/ml, respectively) are suggestive of possibility of either prolonged cycles due to extended luteal phase/delayed luteal regression and/or delayed embryonic death. Significantly higher mean plasma progesterone level (5.58±0.99 ng/ml) recorded on day 7 in CIDR group might be due to the continuous release of the exogenous progesterone from the progesterone molded silastic coil inserted in the anterior vagina of the cows. In the Ovsynch protocol the rise in mean progesterone level (4.10±0.78 ng/ml) noted on day 7 might be due to luteinization of some of the growing follicles and/or ovulation of dominant follicle and formation of accessory CL under the influence of GnRH, simulating diestrum phase, while in the Norgestomet ear implant protocol the mean plasma progesterone level (1.92±0.23 ng/ml) did not show rise in the value probably due to presence of synthetic progestagen in that implant, which is not detected by 17α-hydroxyprogesterone RIA.

### Biochemical and mineral profile

The results of biochemical analysis did not reveal significant variations in plasma total cholesterol, total protein, calcium, and inorganic phosphorus profiles between days and periods of the treatment in any of the groups or between conceived and non-conceived cows. Although the cholesterol concentration was non-significantly higher, and protein was lower in conceived as compared to non-conceived cows. However, the pooled values of cholesterol and protein were significantly higher in normal cyclic cows than in anestrus cows of CIDR and Ovsynch groups (Table-3). Similar results of cholesterol and protein were observed in anestrus cows and buffaloes by earlier researchers [7,26]. However, others [27] reported that the conceiving cows and buffaloes had significantly higher levels of plasma cholesterol and protein as compared to non-conceiving ones. Earlier the higher mean plasma total cholesterol levels at induced estrus and 22^nd^ day post-AI than that of pre-treatment level in GnRH treated anestrus buffaloes have been documented [28], and the high level of cholesterol increased the estrogen synthesis resulting in manifestation of heat [28]. The higher levels of cholesterol in cyclic as compared to acyclic cows and buffaloes are however also reported by previous workers [7,29,30]. Patel *et al*. [7] in crossbred cows, Ramakrishnan *et al*. [25] in Gir cows, and Nakrani *et al*. [5] and Savalia *et al*. [26] in buffaloes,however, recorded significantly higher total protein in conceived than non-conceived and in cyclic than anestrus animals. Gentile *et al*. [31] opined that serum protein level was not related with fertility in dairy cows. However, as has been noted in the present study Patel *et al*. [7] also opined that the crossbred cows having a high level of total protein had good reproductive performance.

**Table-3:** Mean values of plasma protein, cholesterol, calcium and phosphorus concentrations in normal cyclic and anestrus cows under various estrus induction protocols.

Reproductive status	Treatment protocol	Pregnancy status	No.	Days from treatment/AI			

				Total cholesterol (mg/dl)	Total protein (g/dl)	Calcium (mg/dl)	Phosphorus (mg/dl)
Anestrus	CIDR	Conceived	6	129.17±5.45	10.14±0.19	9.08±0.18	4.23±0.19
		Non-conceived	4	111.06±4.04	10.51±0.15	9.40±0.21	3.97±0.14
		Overall	10	121.93±3.88^x^	10.29±0.13^y^	9.21±0.14	4.12±0.13
	Ovsynch	Conceived	5	136.72±1.48	9.30±0.19	9.52±0.14	4.42±0.25
		Non-conceived	5	120.46±5.40	10.80±0.20	8.85±0.11	3.84±0.09
		Overall	10	128.59±3.05^xy^	9.55±0.24^x^	9.19±0.10	4.13±0.14
	Norgestomet ear implant	Conceived	5	132.94±6.35	10.37±0.15	8.84±0.08	3.94±0.11
		Non-conceived	5	137.03±2.14	10.10±0.12	9.62±0.15	4.05±0.09
		Overall	10	134.99±3.33^y^	10.23±0.10^y^	9.23±0.11	3.99±0.07
Normalcyclic	Control	Conceived	4	134.14±8.47	10.78±0.18	9.28±0.26	4.75±0.31
		Non-conceived	6	139.26±4.54	10.97±0.21	9.62±0.30	4.23±0.29
		Overall	10	137.78±4.36^y^	10.61±0.14^y^	9.42±0.24	4.43±0.21

Means bearing uncommon superscripts within the column differ significantly between protocols (p<0.05). The variations between periods and between pregnancy status were not significant in any of the groups, hence not shown here. AI=Artificial insemination, CIDR=Controlled internal drug release

A non-significant influence of CIDR, Ovsynch and/or Norgestomet ear implant protocols in anestrus cows and buffaloes on calcium and phosphorus levels (Table-3) as observed in the present study has also been recently documented by researchers, including normal cyclic control groups [5,7,25,27]. Savalia *et al*. [32] obtained higher mean calcium levels in conceived as compared to non-conceived buffaloes under CIDR, Ovsynch, and even normal cyclic control groups, which is in contrast to the present findings. The marginal deficiency of phosphorus is opined to be enough to cause disturbances in pituitary-ovarian axis, without manifesting specific systemic deficiency symptoms [33]. Savalia *et al*. [32] did not find appreciable variation in the mean plasma inorganic phosphorus levels on the day of GnRH and/or PG treatment, at induced estrus and on day 22 post-AI in anestrus or sub-estrus buffaloes. In the present study, the calcium concentration was non-significantly lower and inorganic phosphorus was higher in conceived than non-conceived cows. This present insignificant differences observed in plasma inorganic phosphorus profile between different phases of the cycle and even conceived and non-conceived groups corroborated with the earlier reports in dairy cows [22,34,35].

## Conclusion

From the results, it can be inferred that the hormonal protocols used, particularly Ovsynch and CIDR protocol, improved conception rates in anestrus crossbred cows under field conditions, and also influenced the plasma progesterone profile significantly, but not the biochemical profile, in a manner of normal cyclic animals, hence can be used by the practicing veterinarians in anestrus rural crossbred cows to improve their reproductive efficiency and thereby the farmers economy.

## Authors’ Contributions

AJD planned and designed the study. The experiment was conducted by AJD, BBN, KKH and JAP, while laboratory work was done by BBN, KKH and RGS. All authors participated in data analysis, preparation of draft of the manuscript, and read and approved the same.
